# Development and validation of a pulmonary function test data extraction tool for the US department of veterans affairs electronic health record

**DOI:** 10.1186/s13104-024-06770-3

**Published:** 2024-04-23

**Authors:** Alexander S. Rabin, Julien B. Weinstein, Sarah M. Seelye, Taylor N. Whittington, Cainnear K. Hogan, Hallie C. Prescott

**Affiliations:** 1 Pulmonary Section, Veterans Affairs Ann Arbor Healthcare System, 2215 Fuller Road, 48105 Ann Arbor, MI USA; 2 Division of Pulmonary and Critical Care Medicine, University of Michigan, Ann Arbor, MI USA; 3 Veterans Affairs Center for Clinical Management Research, Ann Arbor, MI USA

**Keywords:** Pulmonary function test, Electronic health record, Veterans, Forced expiratory volume, Forced vital capacity, Obstruction, Data extraction, Pattern matching, Text mining

## Abstract

**Objective:**

Pulmonary function test (PFT) results are recorded variably across hospitals in the Department of Veterans Affairs (VA) electronic health record (EHR), using both unstructured and semi-structured notes. We developed and validated a hospital-specific code to extract pre-bronchodilator measures of obstruction (ratio of forced expiratory volume in one second [FEV_1_] to forced vital capacity [FVC]) and severity of obstruction (percent predicted of FEV_1_).

**Results:**

Among 36 VA facilities with the most PFTs completed between 2018 and 2022 from a parent cohort of veterans receiving long-acting controller inhalers, 12 had a consistent syntactical convention or template for reporting PFT data in the EHR. Of the 42,718 PFTs identified from these 12 facilities, the hospital-specific text processing pipeline yielded 24,860 values for the FEV_1_:FVC ratio and 23,729 values for FEV_1_. A ratio of FEV_1_:FVC less than 0.7 was identified in 17,615 of 24,922 studies (70.7%); 8864 of 24,922 (35.6%) had a severe or very severe reduction in FEV_1_ (< 50% of the predicted value). Among 100 randomly selected PFT reports reviewed by two pulmonary physicians, the coding solution correctly identified the presence of obstruction in 99 out of 100 studies and the degree of obstruction in 96 out of 100 studies.

**Supplementary Information:**

The online version contains supplementary material available at 10.1186/s13104-024-06770-3.

## Introduction

Pulmonary function tests (PFT) are an essential tool for the assessment of lung disease severity and outcomes in the United States military veteran population. However, PFT reporting in the Department of Veterans Affairs’ (VA) electronic health record (EHR) most commonly occurs in an unstructured or semi-structured format that complicates quantitative and/or qualitative data abstraction and analysis [1]. Prior efforts to extract PFT values (including forced expiratory volume in one second [FEV_1_], forced vital capacity [FVC], and the ratio of FEV_1_:FVC) from VA EHR data sources using natural language processing techniques [1] or automated tools such as a structured query language (SQL) full-text search [2] have focused on select populations [3] or have been limited to measures of FEV_1_ alone [2].

To build on these previously reported methodologies for VA EHR abstraction, we identified VA facilities with a high volume of PFTs performed and applied a site-specific data extraction approach for both quantitative and qualitative reporting of the FEV_1_:FVC ratio and FEV_1_ severity. We then conducted a validation of the abstraction technique, comparing the programming output to manual PFT classification performed by two pulmonary physician adjudicators.

## Methods

### Procedure coding and extraction of notes

PFTs were identified by relevant Current Procedural Terminology codes (94010, 94375, 94060, 94726, 94727, 94729, 94150) for procedures occurring among a cohort of veterans receiving long-acting controller inhalers between January 1, 2018 and December 31, 2022 [4]. Inpatient and outpatient clinical notes from days − 1 to + 21 relative to the PFT date of service were extracted from the VA Corporate Data Warehouse (CDW) [5], a central EHR data repository, using Microsoft SQL Server Management Studio via the VA Informatics and Computing Infrastructure for analysis.

### Identification of semi-structured or unstructured PFT notes containing the FEV_1_ variable

We identified VA facilities with the most PFTs performed during the study period. Among the 36 VA facilities with the most PFTs completed, we assessed the proportion of PFTs completed that had a likely PFT report in the EHR, as evidenced by a clinical node containing the term FEV1. Among facilities with > 80% of PFTs completed having an associated FEV1-containing note, we manually reviewed a random sample of up to 100 notes in JLV to determine whether a consistent approach or template was employed in the semi-structured reporting of PFTs. Unstructured reports (e.g., PFT results reported in physician progress notes) were included only if the notes followed a consistent pattern in the random sample. Reports containing qualitative descriptors of FEV_1_ (e.g., “the FEV_1_ is normal”) were included..

### Creation of a data extraction tool

After identifying high-volume facilities with a consistent approach to PFT reporting in CDW, we developed facility-specific code to extract select PFT results. Each facility-specific code used the following steps: First, the code identified templated PFT result notes based on standard phrasing delineating the start of PFT results. Second, the code extracted a snippet of text from the note (up to 150 characters before and 1000 characters after the appearance of the standard phrase, as shown in Supplementary Fig. 1). Third, the snippet was processed through regular expression pattern matching coding functions to extract the following variables: FEV_1_, FEV_1_ percentpredicted, FEV_1_:FVC ratio, and qualitative reporting descriptors of the PFT results. All coding was performed in Python [6].

### Definitions of spirometric obstruction and FEV_1_ impairment

Obstruction (present or absent) was defined by a threshold of FEV_1_:FVC ratio < 0.7, as suggested by the 2023 Global Initiative for Chronic Obstructive Lung Disease guidelines [7]. FEV_1_ results were mapped to pre-specified percentpredicted values for severity as defined by the 2005 American Thoracic Society/European Respiratory Society (ATS/ERS) PFT interpretation guidelines [8]: normal (FEV_1_ ≥ 80% percent predicted), mildly reduced (FEV_1_ 70–79% percent predicted), moderately reduced (FEV_1_ 60–69% percent predicted), moderately-severely reduced (50–59% percent predicted), severely reduced (35–49% percent predicted), and very severely reduced (< 35% percent predicted). Only pre-bronchodilator values were included.

### Quantitative over qualitative reporting

For the classification of both spirometric obstruction (i.e., FEV_1_:FVC ratio) and FEV_1_ severity, quantitative values were prioritized over qualitative descriptions. However, if the quantitative value was not available, then the qualitative descriptor (e.g., “mild obstruction”) was used.

### Iterative coding process followed by final validation

A random sample of 100 PFT note snippets was selected and reviewed by a pulmonary physician (A.R.) to identify potential coding errors. When the interpretation of the snippet was unclear to the reviewer, an attempt was made to review the original PFT report in the Joint Longitudinal Viewer (JLV), a clinical application allowing read-only access to health data across the VA health system [9]. This iterative process, cross-referencing 100 snippets at a time, was repeated three times to refine the data extraction code.

A random sample of 100 snippets interpreted by the coding solution was used for validation. Two pulmonary physicians (A.R. and H.P.), blinded to the algorithm’s extraction results and to each other’s adjudication decisions, manually recorded the presence or absence of spirometric obstruction and the severity of impairment in FEV_1_ using the previously described criteria. Differences in adjudication were discussed, and consensus was determined in all cases. The consensus adjudications were then compared to the programming output to assess accuracy.

## Results

### VA facility selection for the extraction of PFT reports

**Fig. 1:**
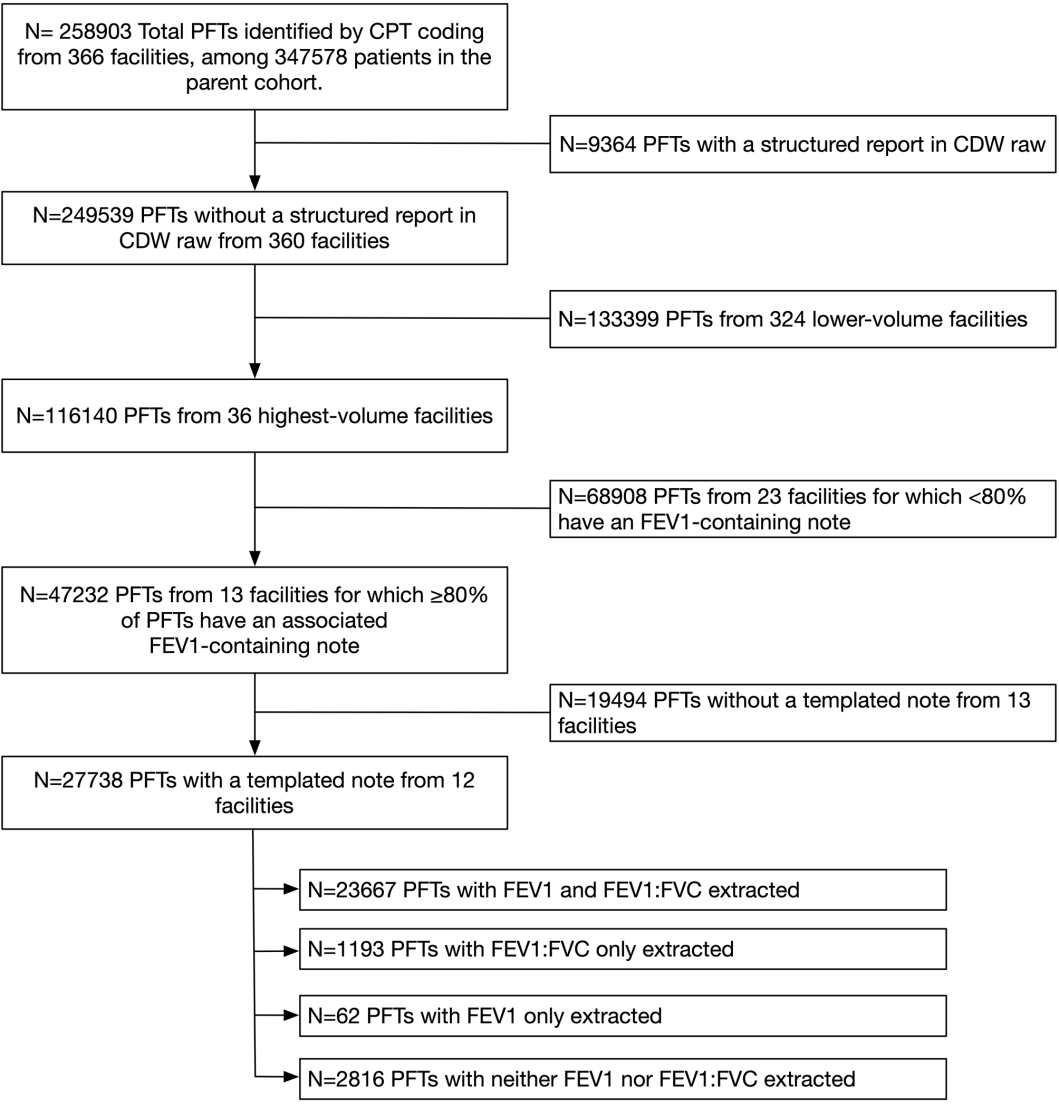
Selection of VA facilities for PFT extraction from the cohort. Flowchart showing the screening process for identification of VA facilities containing semi-structured or unstructured PFT reports. Abbreviations: CDW = corporate data warehouse; FEV_1_ = forced expiratory volume in one second; FVC = forced vital capacity; PFT = pulmonary function test; VA = Veterans Affairs

**Fig. 2:**
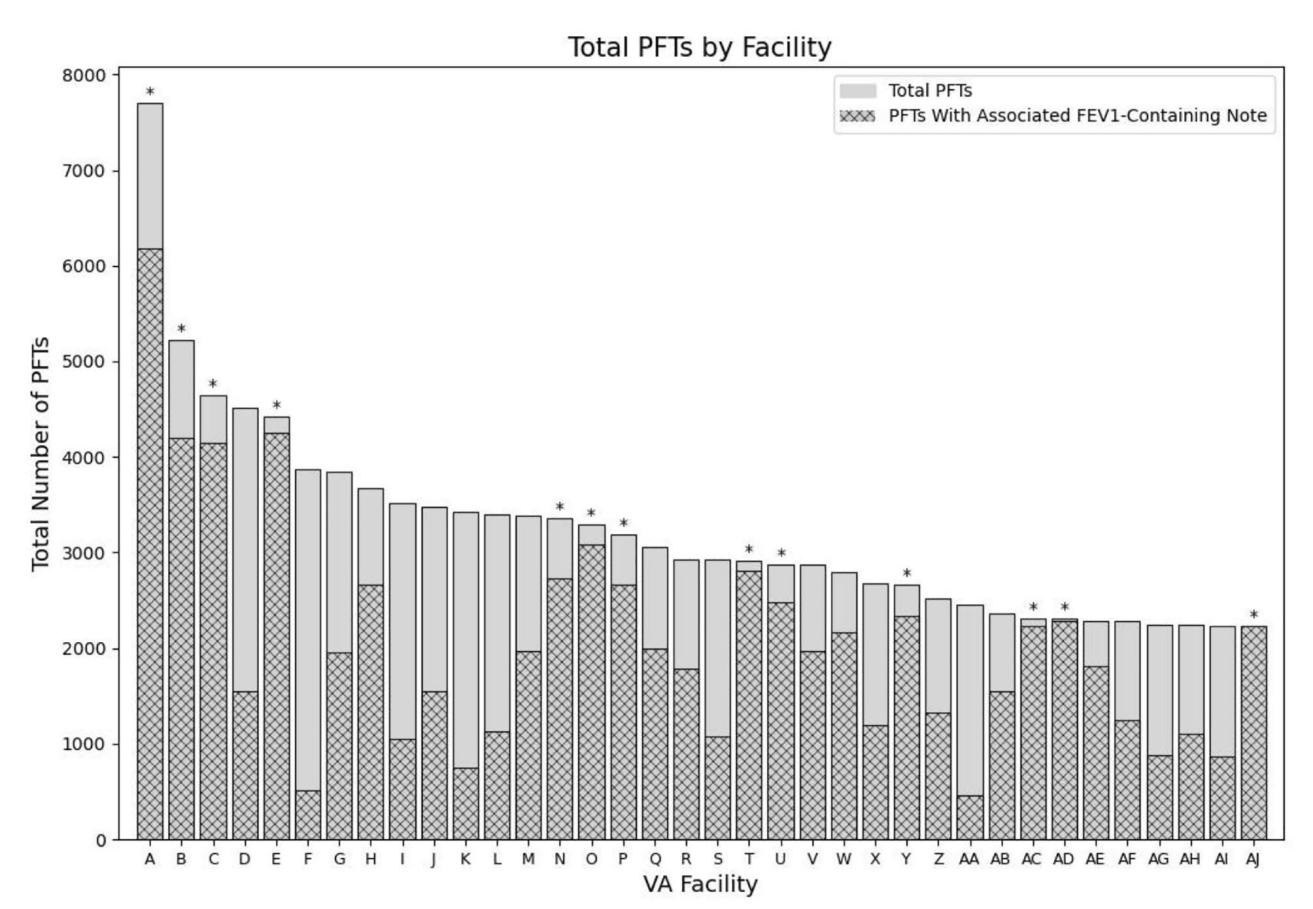
Total and reported PFTs among the 36 highest-volume facilities. Total number of PFTs performed at VA facilities in descending order during the study period. The proportion of PFTs with an associated note containing FEV_1_ are shaded in dark gray. Asterisk (*) indicates facilities with ≥ 80% FEV1-containing notes. Abbreviations: FEV_1_ = forced expiratory volume in one second; PFT = pulmonary function test; VA = Veterans Affairs

Among 347,578 patients receiving long-acting controller inhalers, a total of 258,903 individual PFT studies were identified from 366 VA facilities (Fig. 1). Of these, 9364 (3.6%) studies containing structured FEV_1_ results in CDW Raw were excluded, leaving 249,539 PFTs from 360 facilities for analysis. Of the 36 VA facilities with the most PFT reports, 13 facilities had FEV_1_-containing notes for ≥ 80% of PFTs and underwent further manual review to assess the existence of a standard PFT note template (Fig. 2). One facility with non-standardized reporting of FEV_1_ was excluded.

### Data extraction

Among 42,718 PFT studies from the 12 included facilities, 27,738 PFTs contained an FEV_1_-templated note. A total of 24,860 values for FEV_1_:FVC ratio and 23,729 values for FEV_1_ severity were obtained. The yield of extraction of the FEV_1_:FVC ratio and FEV_1_ values ranged widely across facilities, from 14% for both variables in Facility AJ to 93% for both variables in Facility AD (Supplementary Table 1). The classification of spirometric obstruction and the degree of obstruction are shown in Table 1.

**Table 1:** Classification of obstruction (reduced FEV1:FVC ratio) and degree of FEV1 impairment among PFTs with extracted results

	FEV_1_:FVC ratio			
**FEV**_**1**_ **percent predicted**	≥0.7	<0.7	Missing	Total
Normal	3693	826	16	4535
Mild	974	2418	5	3397
Moderate	758	2897	8	3663
Moderately severe	427	2837	6	3270
Severe	288	5241	18	5547
Very severe	61	3247	9	3317
Missing	1044	149	0	1193
Total	7245	17,615	62	24,922

Cross-tabulation of FEV1:FVC ratio and FEV_1_ results. FEV_1_ categorized as normal (≥ 80% percent predicted), mild (70–79% percent predicted), moderate (60–69% percent predicted), moderately severe (50–59% percent predicted), severe (35–49% percent predicted), or very severe (< 35% percent predicted). Abbreviations: FEV_1_ = forced expiratory volume in one second; FVC = forced vital capacity; PFT = pulmonary function test; VA = Veterans Affairs

### Validation cohort

Among the 100 PFT reports selected for validation, the algorithm correctly graded the presence of obstruction in 99 out of 100 studies. In the same validation cohort, the algorithm correctly assigned FEV_1_ severity, including correctly determining missing FEV_1_, in 96 out of 100 studies.

## Discussion

Access to high-quality lung function data is of paramount importance as the VA seeks to characterize the burden of chronic respiratory disease [10] and explore the long-term effects of airborne hazard exposure on respiratory health [11]. Here we describe a pattern-matching text processing technique for the extraction of semi-structured or unstructured values of FEV_1_:FVC and FEV_1_ from EHR data in a general VA population. This approach could be applied more broadly to the extraction of other PFT variables of interest, including measures of diffusion impairment, lung volumes, or bronchodilator response.

Several prior studies have reported automated extraction of PFT variables from unstructured or semi-structured VA EHR data [1–3]. Using a two-step text processing approach, Akgün et al. showed a high degree of accuracy in the extraction of FEV_1_ values alone from VA progress notes (positive predictive value 99%, 95% confidence interval, 98.2 to 100%) in the Veterans Aging Cohort Study [2]. Another technique using natural language processing to extract FVC values from VA EHR data was accurate, but less applicable to the PFT reporting conventions in CDW beyond the facilities for which it was designed [1].

As the focus of our parent study was on clinical outcomes from inhaler device switching [5], we sought to develop extraction code to best assess for the presence of spirometric obstruction and severity of obstruction while accounting for substantial variability in facility-to-facility PFT reporting conventions. Our approach involved an extensive filtering step that reduced the number of PFT studies supplied to the text mining pipeline; however, the inclusion of longer text snippets and the use of regular expressions in our algorithm allowed for more specific text-matching rules than in previously described methods. The precision afforded by regular expressions to generate customized text processing and extraction procedures at the level of the VA facility resulted in a high degree of accuracy in FEV_1_ value identification.

Strengths of our approach include the use of an adaptable extraction code that enables the text processing of diverse PFT templates across hospitals. These features build on prior methodologies by first identifying high-volume facilities and then refining facility-specific code, thus increasing both the yield and accuracy of the data output. The extensive validation process, involving physician review of hundreds of note snippets cross-referenced with primary EHR data, gives added assurance of data output quality.

In conclusion, this iterative, validated text mining approach for the extraction of PFT data may aid researchers aiming to study pulmonary function housed in unstructured or semi-structured VA data sources.

### Limitations

Our methodology has a number of limitations. First, the code was able to extract FEV_1_ and FEV_1_:FVC ratio for only a minority of PFTs completed in the parent cohort. We developed facility-specific code to increase the yield, but were ultimately limited by the low prevalence of templated notes reporting PFT results. Second, the code was not trained to partition PFT results by date, such as when multiple studies were listed sequentially in a single snippet. In the course of validation, though, this appeared to be an infrequent occurrence. Third, the approach was time- and labor-intensive, requiring multiple revisions and validations in order to maximize data extraction yield and accuracy. Application of the open-source code presented herein could accelerate future efforts to successfully extract PFT variables.

### Electronic supplementary material

Below is the link to the electronic supplementary material.

Supplementary Material 1

Supplementary Material 2

Supplementary Material 3

## Data Availability

No datasets were generated or analysed during the current study. Programming code is available at CCMRPulmCritCare/PFTTextMining (github.com).

## Declarations

### Disclaimer

The views expressed may not represent the position of the U.S. Department of Veterans Affairs or the U.S. government.
