# Supplementary material for: Development and validation of a pulmonary function test data extraction tool for the US department of veterans affairs electronic health record
Source: BMC Res Notes. 2024 Apr 23;17:115. doi: 10.1186/s13104-024-06770-3 (PMC11039415; doi:10.1186/s13104-024-06770-3)
Supplement: Supplementary file 1 — Supplementary Material 1 [file 13104_2024_6770_MOESM1_ESM.docx]

**Supplementary Figure 1: Sample PFT note snippets**

1. PFT report containing quantitative values and a qualitative interpretation


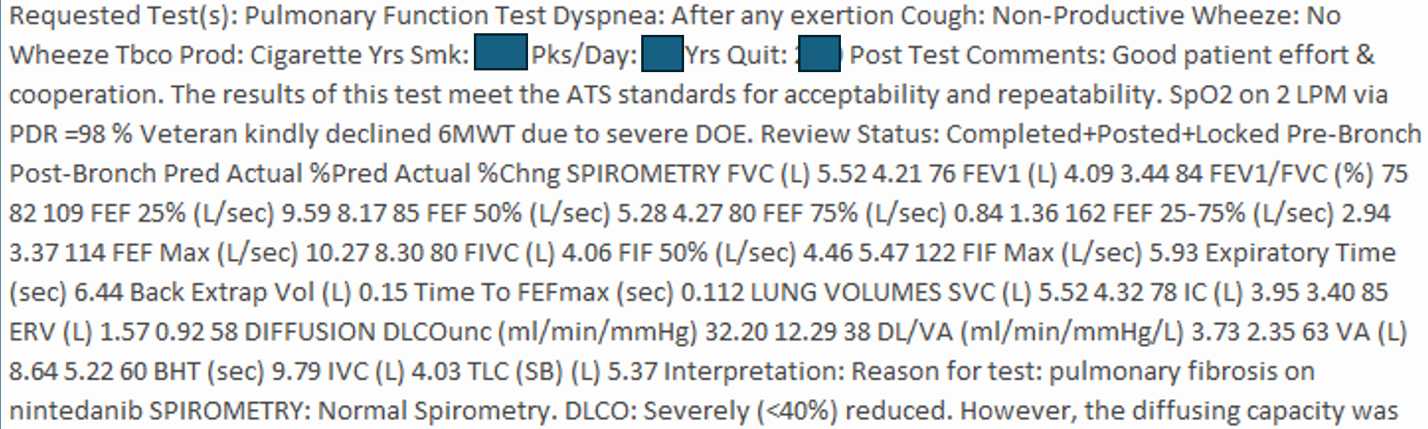


In this example, the pre-bronchodilator ratio of FEV_1_:FVC is 82% (normal) and the pre-bronchodilator FEV_1_ is 84% of the predicted value (normal). The qualitative interpretation of the spirometry is normal.

1. PFT report containing qualitative values


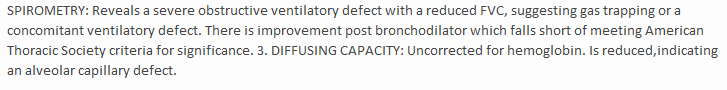


In this example, the qualitative report indicates a severe obstructive ventilatory defect with no quantitative data provided. Abbreviations: FEV_1_ = forced expiratory volume in one second; FVC = forced vital capacity; PFT = pulmonary function test
