# Supplementary material for: Development and validation of a pulmonary function test data extraction tool for the US department of veterans affairs electronic health record
Source: BMC Res Notes. 2024 Apr 23;17:115. doi: 10.1186/s13104-024-06770-3 (PMC11039415; doi:10.1186/s13104-024-06770-3)
Supplement: Supplementary file 2 — Supplementary Material 2 [file 13104_2024_6770_MOESM2_ESM.docx]

**Supplementary Table 1: Extraction of FEV_1_:FVC and FEV_1_ by VA facility**

| **VA**  **Facility** | **Total PFTs identified** | **PFTs with an FEV_1_-containing note for analysis, N (%)** | **PFTs with an FEV_1_-templated note, N (%)** | **PFTs with FEV_1_:FVC ratio extracted, N (%)** | **PFTs with FEV_1_**  **extracted, N (%)** |
| --- | --- | --- | --- | --- | --- |
| A | 7698 | 6174 (80.20%) | 5183 (67.33%) | 5177 (67.25%) | 5182 (67.32%) |
| B | 5227 | 4193 (80.22%) | 4375 (83.70%) | 3361 (64.30%) | 2551 (48.80%) |
| C | 4639 | 4141 (89.26%) | 1377 (29.68%) | 1292 (27.85%) | 1271 (27.40%) |
| N | 3354 | 2729 (81.37%) | 2331 (69.50%) | 1874 (55.87%) | 1874 (55.87%) |
| O | 3291 | 3084 (93.71%) | 3078 (93.53%) | 2684 (81.56%) | 2683 (81.53%) |
| P | 3194 | 2667 (83.50%) | 3013 (94.33%) | 2486 (77.83%) | 2486 (77.83%) |
| T | 2916 | 2803 (96.12%) | 1989 (68.21%) | 1968 (67.49%) | 1960 (67.22%) |
| U | 2878 | 2481 (86.21%) | 1808 (62.82%) | 1513 (52.57%) | 1467 (50.97%) |
| Y | 2669 | 2331 (87.34%) | 775 (29.04%) | 727 (27.24%) | 467 (17.50%) |
| AC | 2309 | 2236 (96.84%) | 1342 (58.12%) | 1327 (57.47%) | 1325 (57.38%) |
| AD | 2309 | 2280 (98.74%) | 2142 (92.77%) | 2142 (92.77%) | 2141 (92.72%) |
| AJ | 2234 | 2227 (99.69%) | 325 (14.55%) | 309 (13.83%) | 322 (14.41%) |
| Total | 42,718 | 37,346 (87.42%) | 27,738 (64.93%) | 24,860 (58.20%) | 23,729 (55.55%) |

Extracted FEV_1_:FVC ratio and FEV_1_ variables by VA facility. FEV_1_:FVC ratio and FEV_1_ counts are a function of the proportion of total PFTs. Abbreviations: FEV_1_ = forced expiratory volume in one second; FVC = forced vital capacity; PFT = pulmonary function test; VA = Veterans Affairs
