# Supplementary material for: Development and validation of a pulmonary function test data extraction tool for the US department of veterans affairs electronic health record
Source: BMC Res Notes. 2024 Apr 23;17:115. doi: 10.1186/s13104-024-06770-3 (PMC11039415; doi:10.1186/s13104-024-06770-3)

**Supplementary Appendix: Pulmonary function test extraction code for one sample VA facility.**

**Full Python programming code available in GitHub at** [CCMRPulmCritCare/PFTTextMining (github.com)](https://github.com/CCMRPulmCritCare/PFTTextMining)

**Step 1: Import packages**


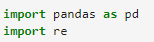


**Step 2: Import dataset and perform initial preprocessing steps**


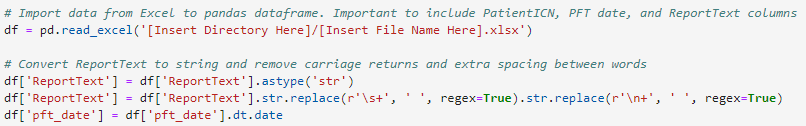


**Step 3: Create function to generate snippets from notes**


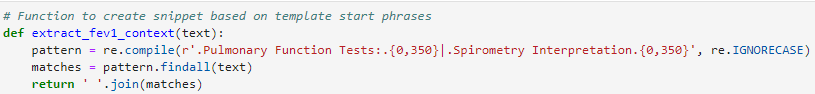


**Step 4: Generate Snippet column by applying the  extract_fev1_context() function to the ReportText column**


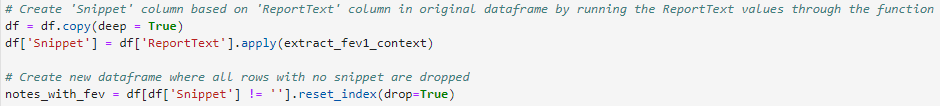


**Step 5: Initialize Function to Classify PFT Results**
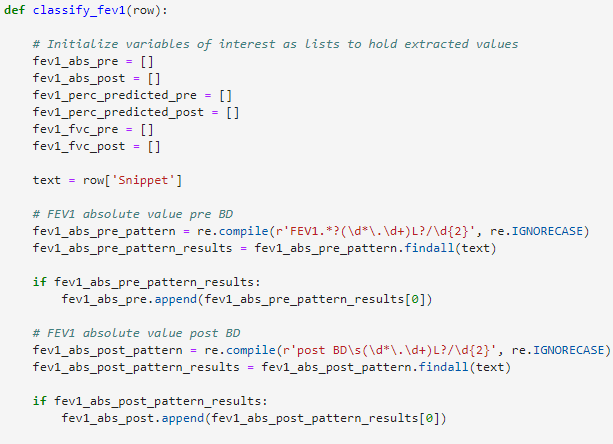


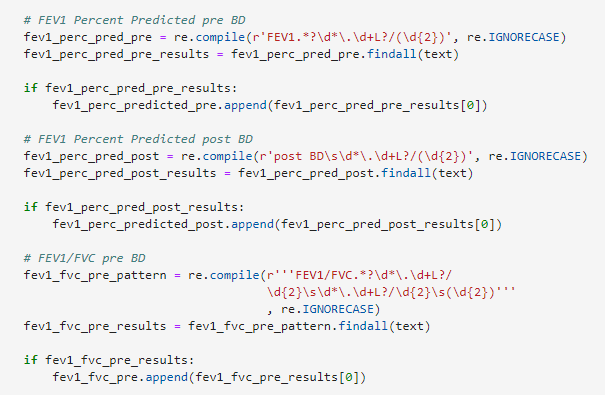


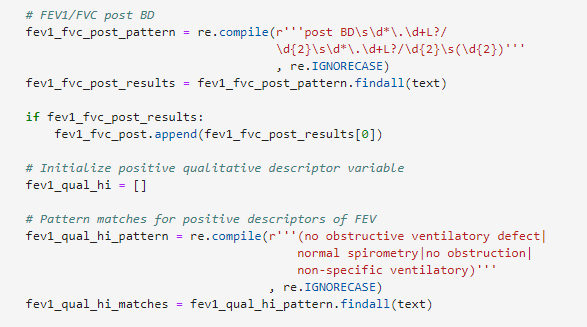


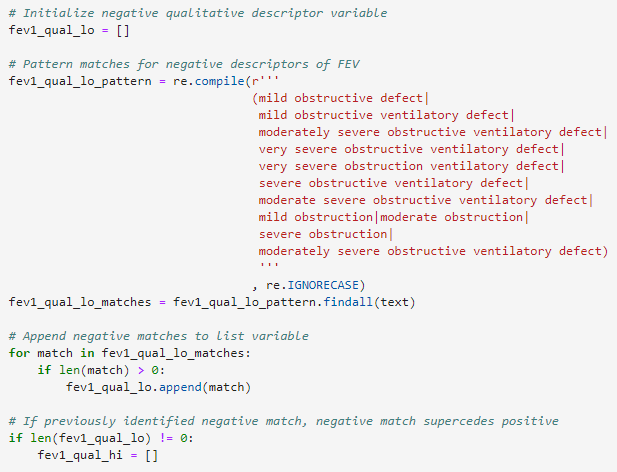


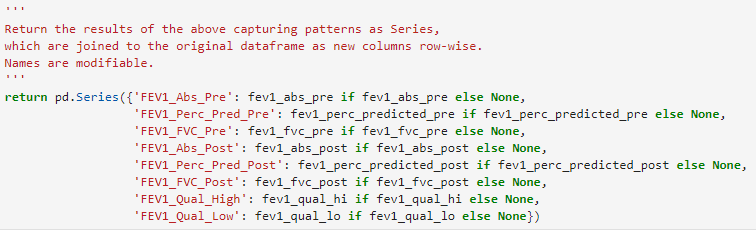


**Step 6: Run data frame through the PFT extraction function**


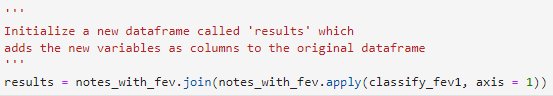


**Step 7: Extract values from FEV1 % predicted, FEV1:FVC pre-BD, and qualitative variables.**


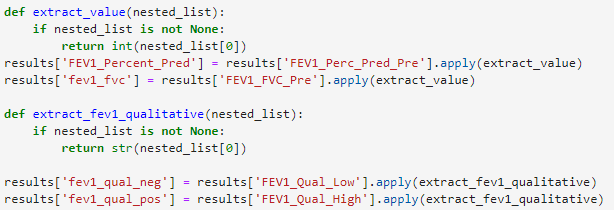


**Step 8: Create mapping functions to map quantitative values to the standard clinical definitions of obstruction and severity of obstruction**


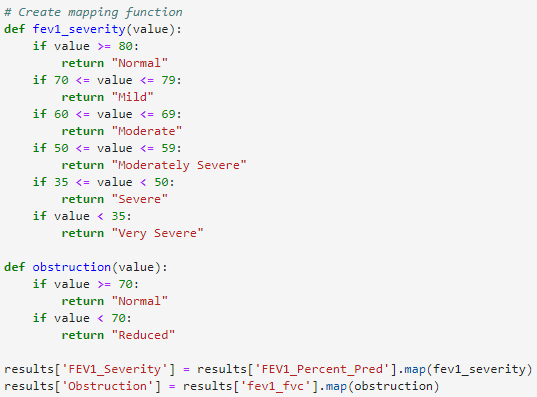


**Step 9a: Impute FEV1 severity values from qualitative data if quantitative FEV1 severity data is absent**
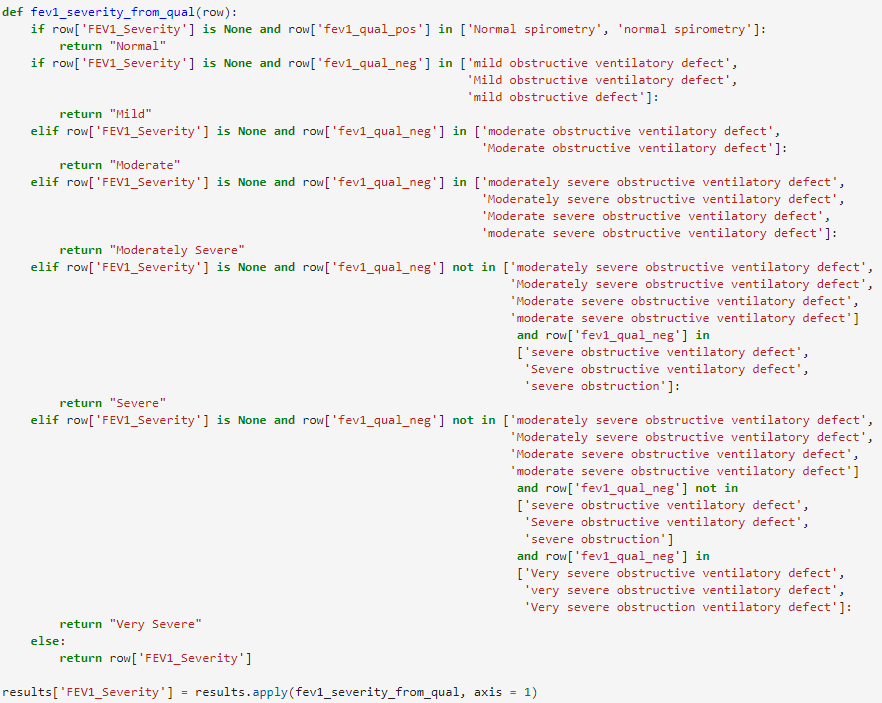


**Step 9b: Impute obstruction values from qualitative data if quantitative obstruction data is absent**


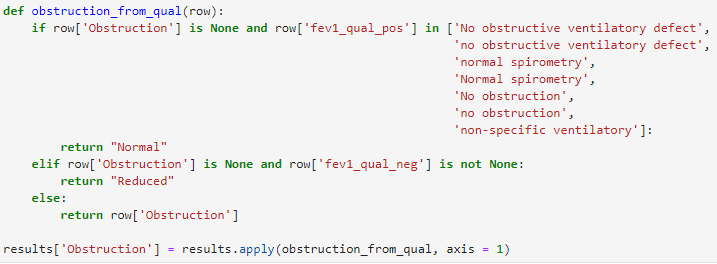


**Step 10: Drop duplicate rows or rows missing extracted PFT data**


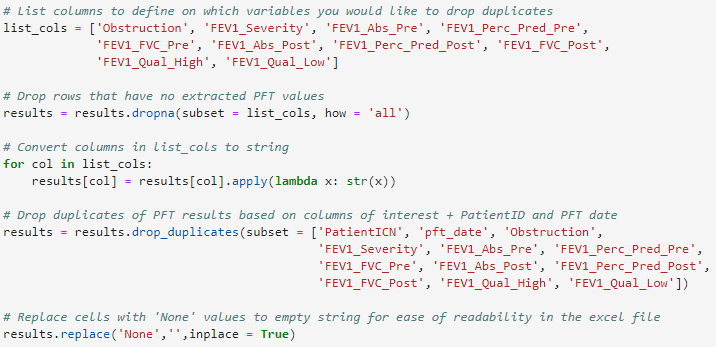


**Step 11: Merge unique data for identical PFTs with different notes**


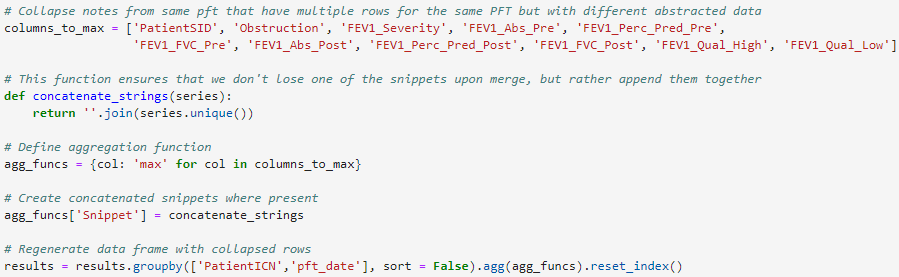


**Step 12: Export data to Excel for validation/analysis**


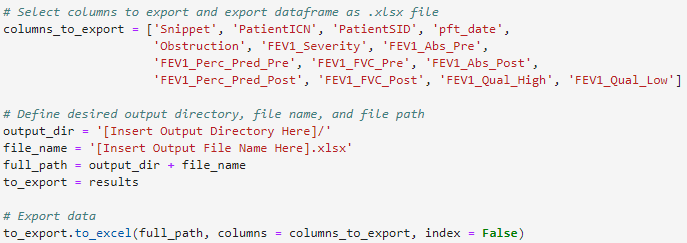

Supplement: Supplementary file 3 — Supplementary Material 3 [file 13104_2024_6770_MOESM3_ESM.docx]
